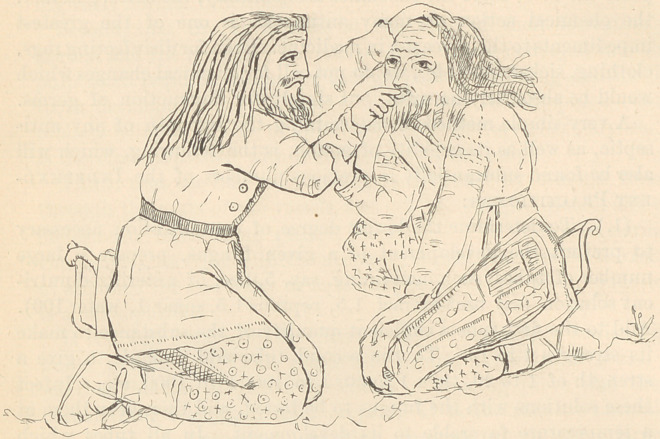# Scythian Dentistry

**Published:** 1886-06

**Authors:** W. H. Eames

**Affiliations:** St. Louis, Mo.


### SCYTHIAN DENTISTRY.

BY W. H. EAMES, D. D. S., ST. LOUIS, MO.

One of the earliest records of a dental operation is found upon a Scythian vase discovered in an immense tumulus or burial mound, situated about four miles to the westward of Kertch, a small town on the Crimean peninsular, at the entrance of the Straits which join the Black Sea with the sea of Azov. Historically, we know but little of the Scythians beyond the meagre facts recorded by Herodotus, but in the almost numberless tumuli which are found upon the Crimean coasts are preserved a most graphic record of their daily lives, manners and customs, in the funereal vases and other objects deposited in the final resting places of their dead.

About the sixth century B. C., a Greek colony from Miletus was founded upon the shores of the Bosphorus, and called Panticapæum. Constant communication was kept up between the colonists and the cities of Greece, and a great and powerful community arose, accumulating vast riches and introducing the unapproachable art of Greece, and adapting it to the uses of the nomadic Scythians as well as the more civilized people who created what is known as Greco-Scythian art.

The richest of the numberless tumuli so far opened is one called the Koul-Oba, which was examined under the direction of the Russian government, and although the greatest care was taken to preserve the precious relics, the larger part was stolen and never recovered. It has been estimated that out of a total weight of one hundred and twenty pounds of solid gold found, the government recovered but fifteen pounds.

The Koul-Oba was a royal tomb, and in a spacious apartment, constructed of large blocks of stone, was found the mouldering remains of a king, his queen or favorite wife, his servants, horses, and surrounded by his treasures. The body of the king lay upon a superb couch composed of massive beams of carved and painted Yew-wood, over which was a canopy. These paintings are purely Greek in character, and are perfectly preserved, notwithstanding the more than twenty centuries which have elapsed since their execution. Near the splendid wooden Sarcophagus of the king were the remains of a woman, doubtless his queen. On her head was a mitre-shaped diadem, and at her feet a small vase of electrum, upon which is embossed a frieze of characteristic episodes of Scythian life. Electrum, an alloy composed of gold with a fifth part silver, was highly valued by the Greeks, its color being paler and more luminous than gold. Upon the vase are four groups in exquisite repousse work, giving incidents in the life of the same person. The king is clad in the Scythian costume, a tunic belted at the waist, and full trousers tucked in his boots; which is almost identical with the Russian costume of to-day. In one group he is listening to the report of a warrior kneeling before him, in another he is bending a bow, in the third his wounded leg is being dressed by an attendant, and the last, as before stated, is one of the earliest known representations of an operation in dentistry. The king is half sitting, half kneeling, while the Scythian dentist is extracting a tooth from the left side of the jaw. It is reasonable to suppose that this represents an actual incident in the life of the skeleton found in this tomb. In the skull, now deposited in the museum at Kertch, the first and second left lower molars are missing, and the third molar is badly decayed. The presence of an alveolar abscess connected with these lost teeth at some period of life is shown by the condition of the alveolar process in this region.

The only clue to the identity of the powerful monarch here entombed is an inscription of three letters upon an ornament, in which it is claimed by some that they recognize the initials of a Bosphorus King, Pairisades, the son of Satyrus, who reigned about 310, B. C.

In 1880, Earl Granville, British Secretary of State for Foreign Affairs, brought before the Russian Ambassador to London the request of the Committee of the Council on Education, to secure for the South Kensington Museum, copies of some of the numerous examples of Russian art contained in the Imperial collections of Russia. The request was readily granted, and many beautiful selections were made for reproduction. These have recently been completed and placed at South Kensington, and a most complete handbook of Russian art has been published, which is intended not only as a guide-book to the Museum, but to give general information upon a subject heretofore but little known. It is from this work that the substance of this article has been derived.

## Figures and Tables

**Figure f1:**